# Epigenetic controls of Sonic hedgehog guarantee fidelity of epithelial adult stem cells trajectory in regeneration

**DOI:** 10.1126/sciadv.abn4977

**Published:** 2022-07-22

**Authors:** Fanyuan Yu, Feifei Li, Liwei Zheng, Ling Ye

**Affiliations:** ^1^ State Key Laboratory of Oral Diseases and National Clinical Research Center for Oral Diseases, West China Hospital of Stomatology, Sichuan University, Chengdu, China.; ^2^ Department of Endodontics, West China Hospital of Stomatology, Sichuan University, Chengdu, China.; ^3^ Department of Pediatric Dentistry, West China Hospital of Stomatology, Sichuan University, Chengdu, China.

## Abstract

Given that adult stem cells (ASCs) fuel homeostasis and healing by providing tissue-specific descendants, the fidelity of ASC fate determination is crucial for regeneration. Here, we established that an epigenetic control of epithelial ASC fate fidelity via Ezh2/H3K27me3 was indispensable for incisor homeostasis and regeneration. Mechanistically, in homeostasis, H3K27me3 upstream occupies the Sonic hedgehog (Shh) promoter to directly restrain Shh expression, thereby precisely confining Shh expression. When injury occurred, Ezh2/H3K27me3 was substantially induced within inner enamel epithelium and preameloblast zones, and such epigenetic response guaranteed the fidelity of ASC commitment via pulling injury-increased Shh back to homeostatic levels, utterly underlying regeneration progression. Once losing H3K27me3-dependent restriction of Shh expression through the Cre-Loxp system totally disrupted lineage commitment and stemness exhaustion, and abolished hard tissue regeneration emerged in vivo. We next uncovered the molecular mechanisms by which injury-induced Ezh2 mediated the spatiotemporal dynamics of H3K27me3 to repress Shh expression, thus epigenetically deciding ASC fate.

## Teaser

Injury-induced epigenetic signal of Ezh2/H3K27me3 guaranteed fidelity of epithelial ASC fate decision via restraining Shh.

## INTRODUCTION

Fidelity of orchestrating stem cells fate underlies tissue regeneration (*1*, *2*). Adult stem cells (ASCs) can provide mitotic cells and postmitotic committed descendants to replace lost tissues and meanwhile maintain stem cell population (*3*, *4*). By these primary mechanisms, ASCs enable injury healing and the rebuilding of tissue homeostasis (*1*, *3*). Disrupting precision of ASC fate determination often leads to insufficient lineage commitment and exhaustion of stemness reservoir, utterly impairing tissue regeneration (*1*–*4*). Therefore, understanding the mechanisms of injury-triggered ASC fate determination will endow us with better solutions to boost adult tissue regeneration.

As a fully regeneration capable epidermal appendage, rodent incisors provide an ideal model to investigate fate choice of ASCs (*5*–*8*). In detail, epithelial and mesenchymal ASCs, residing at the proximal end of incisors, can give rise to all cell types of adult teeth, including ameloblast, cementoblast, and odontoblast lineages, thus empowering the replacement of lost tissues resulting of abrasion or injury (*5*–*8*). Via using a rodent incisor model, researchers have found an essential regulator for mesenchyme-derived hard tissue regeneration, which was Sonic hedgehog (Shh) secreted from neurovascular bundles (NVBs) (*7*). Mechanistically, NVB-derived Shh dictated mesenchymal stem cell differentiation to regenerate lost dentine (*7*). There exists another Shh signal hub in adult incisors, which was localized at the transient amplifying (TA) region of epithelium, including inner enamel epithelium (IEE) and preameloblast (PAB) (*9*). However, the functional roles of epithelium-derived Shh in adult enamel regeneration are poorly elucidated.

Shh, the principal ligand of Hedgehog (Hh) pathway, has been proposed to play key roles in tooth germ development, including regulation of epithelial cell proliferation, growth, polarization, and so on (*10*–*13*). In adult mouse incisors, Shh was proved to be indispensable for amelogenesis because inhibition of Hh signaling pathway via small molecules ceased the generation of committed ameloblasts (*9*). These data together strongly suggested that epithelium-derived Shh might be a vital signal to orchestrate cell trajectory of dental epithelial stem cells (DESCs). Here, in this study, we sought to furthermore elaborate on the relationship between Shh and epithelial ASC-mediated hard tissue regeneration.

In addition to the unclear role of epithelium-derived Shh in regeneration, the upstream regulatory mechanisms of Shh expressions in incisor epithelium yet remain totally unknown (*14*). Taking the highly orchestrated spatial architecture of Shh within TA and PAB zones and the decisive function of Shh for TA and PAB cells into consideration, it can be assumed that there should be an upstream regulator with high sensitivity and fidelity in epithelium to control Shh expression in a precisely spatiotemporal pattern (*9*, *14*). Intriguingly, in nerve systems, polycomb-repressive complex 2 (PRC2)–mediated H3K27me3 has been reported to be such kind of upstream decider of Shh expression in schwann cells (*15*). This work demonstrated that H3K27me3 highly occupies within the transcript start region of Shh, thus repressing its transcription (*15*). Besides, our previous study also showed that Shh-resident regions were weakly modified by H3K27me3, suggesting that H3K27me3 may also play an upstream restraining role for Shh expression in incisor epithelium (*16*). As a repressive histone modification, H3K27me3 has been proposed to regulate self-renewal, lineage specification, and survival of diverse kinds of stem cells, indicating its indispensable functions on stem cell fate determination (*17*–*20*). However, little is known about the functional role of H3K27me3 in ASC-mediated organ regeneration (*21*–*23*).

To address the aforementioned issues, in this study, we hypothesized that H3K27me3 may be involved in adult incisor regeneration through Shh-mediated ASC fate determination. Specifically, we comprehensively investigated the upstream epigenetic mechanisms that controlled Shh expression in incisor epithelium and whether such upstream regulation of Shh can influence epithelium-derived hard tissue regeneration. Through exploring these aspects, we depicted a cellular and molecular atlas of controlling the fidelity of adult DESC fate determination during organoregeneration and injury responses. Our findings will endow researchers with thorough scopes to further comprehend the role of ASC fate determination in tissue homeostasis and regeneration.

## RESULTS

### Injury-induced cell fate alterations in adult incisor epithelium

**Fig. 1.**
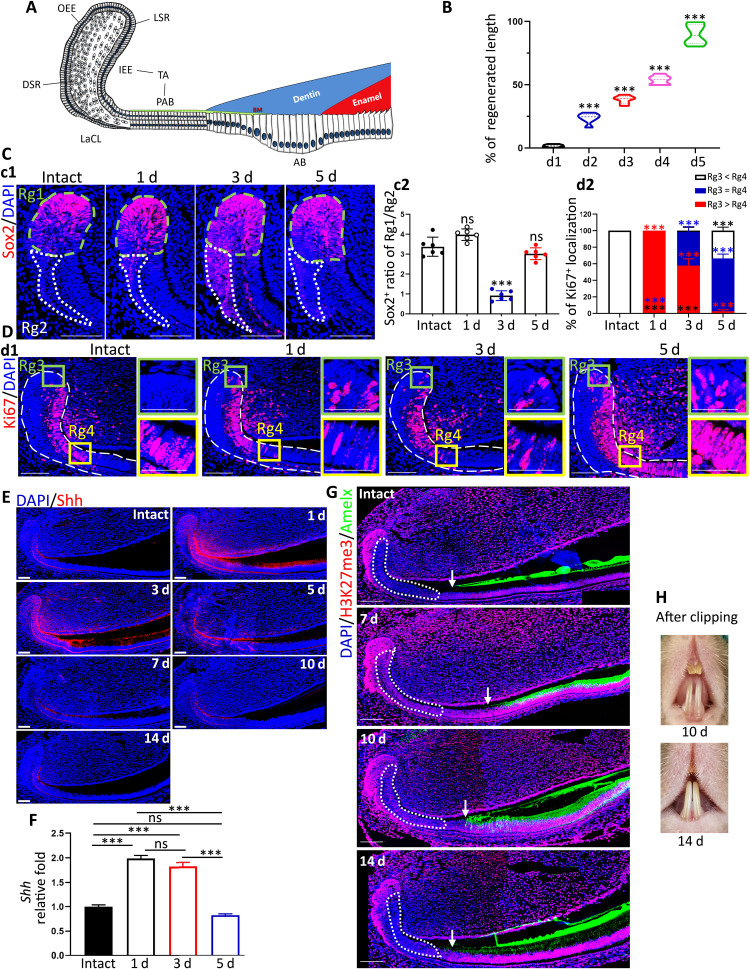
Identification of injury-induced cell fate alterations and spatiotemporal expression of Shh and H3K27me3 status. (**A**) Schematic illustration of dental epithelium of adult mandibular incisors. (**B**) Statistical data of the regeneration ratio of mandibular incisors at different time points after clipping at the gingival level. (**C**) c1: Immunofluorescent images of Sox2^+^ DESCs in LaCL of intact or defect incisors. c2: The ratio of Sox2^+^ DESCs in the upper layer of LaCL over that at the bottom of LaCL at different time points after injury. (**D**) d1: Immunofluorescent images of Ki67^+^ proliferative cells. d2: Statistical data of d1. (**E**) Representative IF images of Shh. The labels of days indicated the days after clipping. (**F**) Statistic RT-qPCR data of Shh in healthy and different stages after clipping. (**G**) Representative IF images of H3K27me3 and Amelx in intact condition and 7, 10, and 14 days after clipping. Dotted areas indicate the IEE region. The arrow indicates the deposit start site of Amelx. (**H**) Representative photographs of incisors at 7 and 10 days after clipping. All representative images and data (means ± SEM) were generated from at least six slices from five mice for each group. ns, no statistical significance; *P < 0.05, **P < 0.01, and ***P < 0.001. The dot-square contoured regions were shown in the right as high-magnification images. OEE, outer enamel epithelium; DSR, dense stellate reticular layer; LSR, loose stellate reticular layer; BM, basal membrane; AB, ameloblasts. Scale bar, 50 μm.

To investigate how incisor epithelial lineages respond to injury, we used an incisor clipping model. Adult mouse incisors clipped at the gingival margin could be fully regenerated within 2 weeks (Fig. 1, B and H, and fig. S1A). Because Sox2, Ki67, and Amelogenin (Amelx) are well-recognized markers of DESCs, proliferating progenitors, and differentiated ameloblasts, respectively (*8*, *24*, *25*), we set out to study the behavior of these three different cell clusters by immunofluorescence (IF) stainings during incisor regeneration. We found that Sox2 was abundantly expressed in the upper layer of a labial cervical loop (LaCL) (Rg1) while much less expressed at the bottom (Rg2) on intact condition (Fig. 1C). After incisor clipping, Sox2^+^ cells in Rg1 and Rg2 were increased at day 5 (d5) and d3, respectively, which resulted in Sox2^+^ ratio of Rg1/Rg2 markedly decreased on d3, and returned to the homeostatic level on d5 (Fig. 1C). We observed that the increment of Sox2^+^ cell in Rg1 obviously lagged behind that in Rg2 after clipping, which was assumed to supply stem cells required for regeneration. However, the verification of this hypothesis calls for additional lineage-tracing data. As to proliferating progenitors, we observed that Ki67^+^ cells were maximally located at TA and PAB zones (Rg4) but not at the top of LaCL (Rg3) on intact condition. After clipping, Ki67^+^ cells emerged at Rg3 on d1 and lasted until d5. In contrast, Ki67^+^ cells disappeared at Rg4 on d1 and reappeared at d3 and d5 (Fig. 1D). These results suggested that Ki67^+^ cells at Rg3 might supplement the consumed proliferating cells at Rg4 during regeneration. We next detected Shh expression after clipping. Results showed that injury significantly increased the Shh level in incisor at 1 day after clipping (Fig. 1E). At d3 and d5 after clipping, the Shh level was starting to decline in comparison to that at d1, and then at d7, d10, and d14 after clipping, the expressions of Shh were the same as that in intact condition (Fig. 1E). Furthermore, real time quantitative polymerase chain reaction (RT-qPCR) data of incisor epithelium demonstrated the identical expression pattern of Shh mRNA (Fig. 1F), similar to the IF data (Fig. 1E). Besides, our data showed the onset of Amelx expression moved distally to LaCL at 7 days after injury (Fig. 1G), but at d10 and d14 after clipping, the onset of Amelx expression went back to the homeostatic site as intact incisors (Fig. 1G). Taking the results that incisor achieved the morphologically full regeneration at 10 and 14 days after clipping together (Fig. 1H), our data indicated that, at 10 and 14 days after clipping, the homeostasis began to be rebuilt after injury. In summary, after clipping, the incisor epithelium was regenerated via recruiting more Sox2^+^ progenitors from the bottom to the upper layer of LaCL and via triggering DESC proliferation to supply more descendants, as well as via delaying the onset of differentiation (*5*). Because H3K27me3 is repressive to gene transcription, low H3K27me3 modification in IEE on intact condition was supposed to be in favor of Shh expression, while elevated H3K27me3 in injury was supposed to pull injury-induced Shh back to intact condition (Fig. 1, E to H, and fig. S2, A and B).

### Targeting PRC2 complex abolished epithelium-derived enamel regeneration

**Fig. 2.**
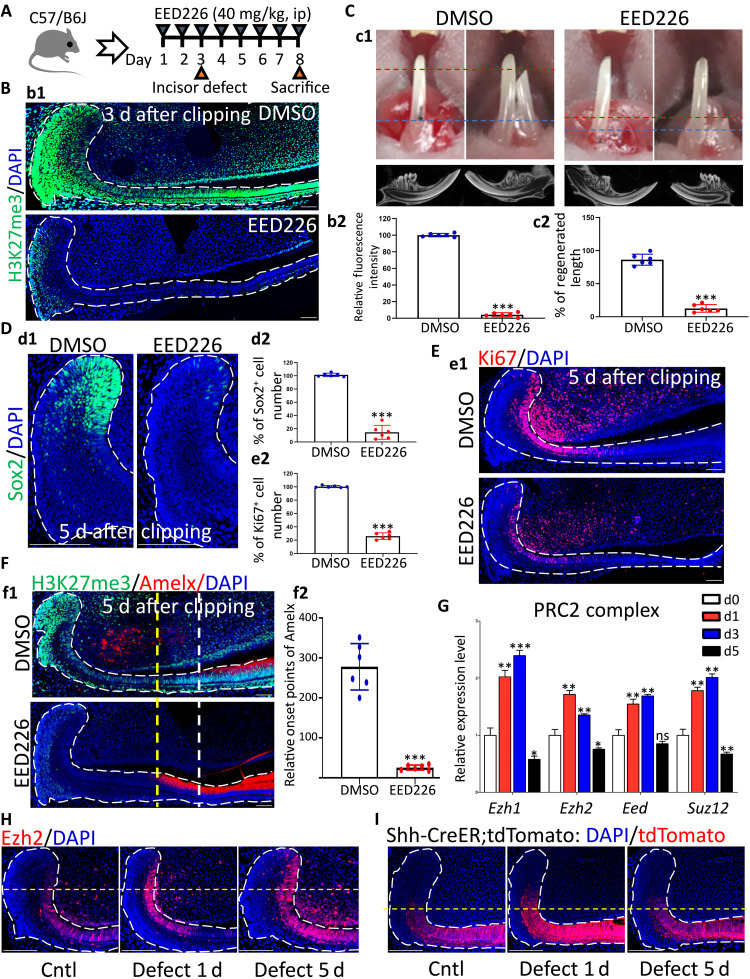
In vivo blocking H3K27me3 through targeting PRC2 complex disrupted injury-induced cell fate alterations and impaired epithelium-derived enamel regeneration. (**A**) Schematic illustration of EED226 administration plan and the strategy to establish incisor defect models; ip, interperitoneal injection. (**B**) b1: Immunofluorescent images of H3K27me3 in incisors receiving dimethyl sulfoxide (DMSO) or EED226 at d3 after clipping. b2: Statistical data of b1 on the fluorescence intensity of H3K27me3 in dental epithelium. (**C**) c1: Photograph and μCT three-dimensional (3D) reconstruction images of incisors receiving DMSO or EED226 at d5 after clipping. c2: Statistical analysis on regenerated incisor length in c1. (**D**) d1: Immunofluorescent images of Sox2^+^ DESCs in LaCL at d5 after clipping. d2: Statistical data on the number of Sox2^+^ DESCs in d1. (**E**) e1: Immunofluorescent images of Ki67^+^-proliferative cells in dental epithelium at d5 after clipping. e2: Statistical data on the number of Ki67^+^ cells in e1. (**F**) f1: Double-staining immunofluorescent images of H3K27me3 and Amelx in epithelium at d5 after clipping. f2: The relative distally moved distance about the onset point of Amelx expression in f1. (**G**) RT-qPCR data of core subunits of PRC2 complex in LaCL at different time points after incisor clipping. (**H**) Immunofluorescent images of Ezh2 in dental epithelium of intact or defect incisors at different time points. (**I**) Immunofluorescent images of Shh^+^ dental epithelial cells and their descendants at different time points after injury in Shh-CreER;tdTomato mice. All representative images and data (means ± SEM) were generated from at least six slices from six mice for each group. RT-qPCR was repeated three times with three duplicates for each group in each experiment. *P < 0.05, **P < 0.01, and ***P < 0.001. Scale bar, 50 μm.

To investigate the functional requirements of H3K27me3 in incisor regeneration, we blocked H3K27me3 through intraperitoneal injection of EED226 2 days before clipping in adult mice (Fig. 2A). EED226, a small-molecule inhibitor of PRC2, could directly bind to EED, leading to loss of function of the PRC2 complex (*26*). IF data showed effectiveness and specificity of EED226 because it blocked H3K27me3 both in incisor mesenchyme and epithelium but did not alter other epigenic modification such as H3K9me3 (Fig. 2B and fig. S2F). Gross images and micro–computed tomography (μCT) consistently revealed markedly reduced enamel regeneration in the EED226 injection group, suggesting an essential role of H3K27me3 in incisor hard tissue regeneration (Fig. 2C). Furthermore, IF staining showed that EED226 remarkably decreased cell numbers of Sox2^+^ DESCs and Ki67^+^-proliferating progenitors and moved the onset of Amelx expression proximally to LaCL at d5 after injury (Fig. 2, D to F). To avoid the possible effects on homeostasis caused by PRC2 inhibition prior to injury, we changed the administration strategy via injecting EED226 at d1 after clipping. Severely impaired incisor regeneration could also be observed in this model of EED226 administration (fig. S2, C to E). Together, these data demonstrated that loss of H3K27me3 interrupted injury-induced cell fate alterations, thus leading to damaged regeneration capability of incisor epithelium.

### Depleting Ezh2 in Shh^+^ lineage caused dyshomeostasis of epithelial ASCs

**Fig. 3.**
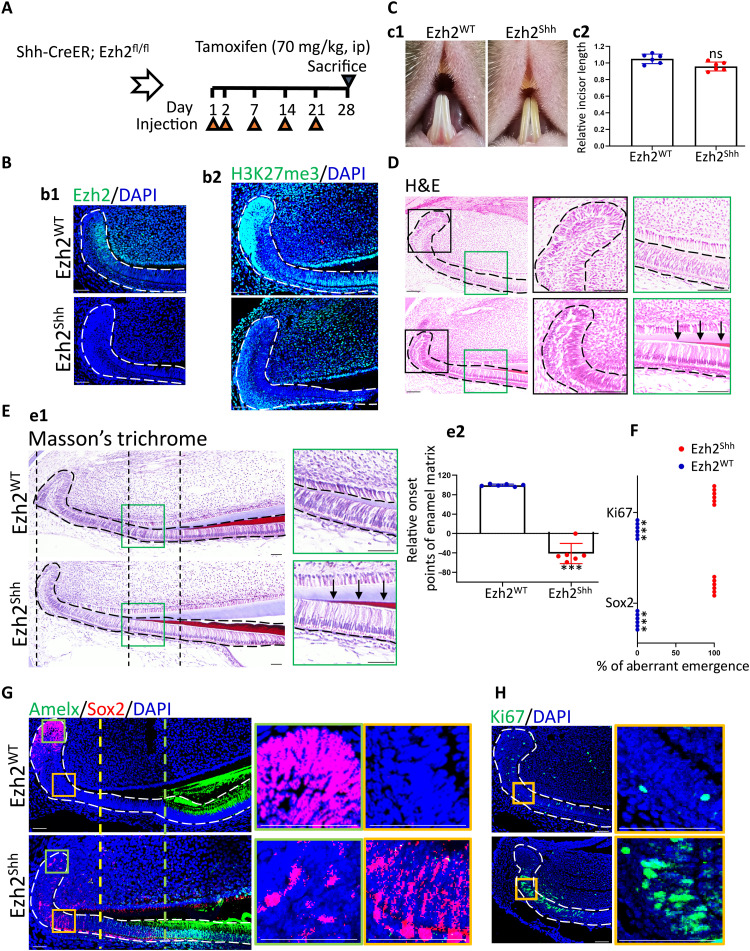
Depletion of Ezh2 in Shh^+^ lineage led to dyshomeostasis of mandibular incisors and disruption of epithelial cell fate. (**A**) Schematic illustration on tamoxifen administration plan of Shh-CreER; Ezh2^fl/fl^ (Ezh2^Shh^) or Ezh2^fl/fl^ control littermate (Ezh2^WT^) mice. (**B**) Immunofluorescent images of Ezh2 and H3K27me3. (**C**) c1: Photograph images of Ezh2^Shh^ or Ezh2^WT^ incisors after 1-month tamoxifen administration. c2: Statistical analysis on incisor length in c1. (**D**) H&E images. The arrows in the high magnification indicate precolumnized ameloblasts and predeposited dark eosinophilic matrix in Ezh2^shh^ incisors. (**E**) e1: Masson’s trichrome images. The arrows in the high magnification indicate enamel matrix that was dyed red. e2: Statistical analysis on the location of onset points of enamel matrix in e1. (**F**) Statistical analysis of (E) on the ratio of aberrant emergence. (**G**) Double-staining immunofluorescent images of Sox2 and Amelx. (**H**) Immunofluorescent images of Ki67. All representative images and data (means ± SEM) were generated from at least six slices from six mice for each group. ***P < 0.001. The dot-square contoured regions were shown in the right as high-magnification images. Scale bar, 50 μm.

Because abolished enamel regeneration in the EED226 injection group could be attributed to overall blockade of H3K27me3 in mesenchyme and epithelium, to specifically investigate the role of responding increment of H3K27me3 in TA and PAB regions after injury, we genetically deleted Ezh2 in Shh^+^ epithelial lineage. Because Ezh2, the major catalytic subunit of PRC2 complex, was the earliest responding subunit of this complex after incisor clipping, specifically, Ezh2 expression reached the summit only at 1 day after injury, while other subunits of PRC2 complex did so at d3 after clipping (Fig. 2G). Meanwhile, injury-induced Ezh2 and Shh spatiotemporally overlapped a lot in IEE and PAB regions, indicating the availability of Shh-CreER, instead of currently common Cre tools of incisor epithelium such as K14, to specifically erase injury-induced Ezh2 activity (Figs. 1G and 2H). In detail, according to our data, both Ezh2 and Shh were expressed in IEE and PAB within intact incisor epithelium but mutually extended their expression to the upper layer of LaCL after injury (Figs. 1G and 2H). On the basis of this, in following investigations, we crossed an Ezh2 conditional allele (Ezh2^fl/fl^) with Shh^CreER^, in which tamoxifen can induce Cre recombinase activity in Shh^+^ lineage (Fig. 2I and fig. S1B), thus generating Shh^CreER^; Ezh2^fl/fl^ conditional knockout (cKO) mutants (Ezh2^shh^). In addition, the wild-type littermates, Ezh2 ^fl/fl^, were set as controls (Ezh2^WT^). We first ascertained the efficiency and specificity of Ezh2 KO in vivo. Tamoxifen administration strategy was shown in Fig. 3A. IF data showed that Ezh2, as well as H3K27me3 rather than other histone modificatons such as H3K9me3, was successfully depleted in the TA and PAB regions (Fig. 3B and fig. S2F). We next examined the general phenotypes of mandibular incisors 1 month after tamoxifen injection. Ezh2^shh^ incisors were slightly shorter than controls but without statistical significance (Fig. 3C). Hematoxylin and eosin (H&E) staining showed disorganized architecture of LaCL, including thinner outer enamel epithelium (OEE) and small crackles, as well as precolumnization of ameloblasts and predeposition of dark eosinophilic matrix in Ezh2^shh^ incisors (Fig. 3D). To ascertain that the predeposited matrix observed above was enamel matrix, we performed Masson’s trichrome staining (MTS), in which dentine will be dyed as blue while enamel as red. Data showed that the onset of enamel matrix deposition did move proximally to LaCL in Ezh2^shh^ incisors (Fig. 3, E and F).

To understand the cellular events underlying these phenotypes, we accordingly detected Sox2, Ki67, and Amelx in vivo. Data showed that Sox2^+^ DESCs in cKO epithelium were no longer restricted to LaCL, like when they were in WT epithelium, but extended to PAB region (Fig. 3G). In addition, cKO epithelium showed proximally moved onset of Amelx expression, leading to partial overlapping of Sox2 and Amelx in the PAB region (Fig. 3G). It was also in the PAB region that the numbers of Ki67^+^-proliferating progenitors remarkably increased (Fig. 3H). Thus, Ezh2 depletion led to aberrant colocalization of Sox2^+^, Ki67^+^, and Amelx^+^ cells in the PAB region, suggesting severely disrupted cell fate.

### Depleting Ezh2 in Shh^+^ lineage abrogated enamel regeneration

**Fig. 4.**
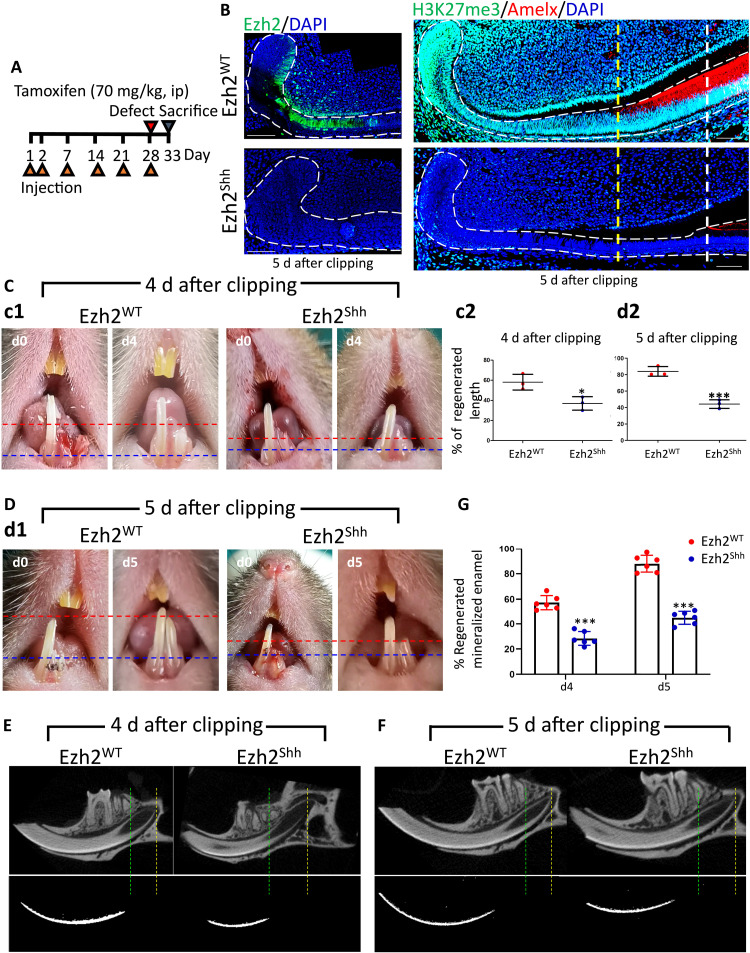
Depletion of Ezh2 in Shh^+^ lineage blocked injury-induced increment of H3K27me3 and abrogated enamel regeneration. (**A**) Schematic illustration on plans of tamoxifen administration and incisor defect establishment using Ezh2^Shh^ or Ezh2^WT^ mice. The following data were described by days after clipping but not the timeline schematic. The detailed information was added in the figure legend. (**B**) Immunofluorescent images of Ezh2, H3K27me3, and Amelx in incisors epithelium of Ezh2^Shh^ or Ezh2^WT^ mice at d5 after clipping. (**C**) c1: Photograph images of Ezh2^Shh^ or Ezh2^WT^ incisors at d4 after clipping. c2: Statistical analysis on the percentage of regenerated incisor length in c1. (**D**) d1: Photograph images of Ezh2^Shh^ or Ezh2^WT^ incisors at d5 after clipping. d2: Statistical analysis on the percentage of regenerated incisor length in d1. (**E**) 3D reconstruction images of μCT on mandibular incisors and mineralized enamel at d4 after clipping. (**F**) 3D reconstruction images of μCT on mandibular incisors and mineralized enamel at d5 after clipping. (**G**) Statistical comparison on the percentage of regenerated mineralized enamel in (E) and (F). All representative images and data (means ± SEM) were generated from at least six slices from six mice for each group. *P < 0.05 and ***P < 0.001. Scale bar, 50 μm.

As our primary aim was to investigate the functional role of Ezh2/H3K27me3 in incisor regeneration, we thus used an Ezh2^Shh^ mutant mouse model to block injury-induced Ezh2/HK27me3 increment. Tamoxifen administration and incisor clipping strategies are shown in Fig. 4A. Data showed no Ezh2 expression and H3K27me3 modification within TA and PAB regions of Ezh2^Shh^, verifying successful and specific depletion of injury-induced increment of Ezh2/H3K27me3 (Fig. 4B). Following photographs and statistical analysis showed that depletion of Ezh2/H3K27me3 caused severely impaired incisor regeneration by 4 and 5 days after incisor clipping (Fig. 4, C and D). Because dentine and enamel could not be distinguished in the photographs, we thus used μCT to accurately exhibit enamel. Data consistently showed apparently delayed enamel mineral deposition and reduced enamel regeneration in Ezh2^Shh^, proving depletion of Ezh2 in Shh^+^ lineage-abrogated enamel regeneration (Fig. 4, E to G).

### Severely disrupted cell fate in Ezh2^KO^ Shh^+^ lineage during enamel regeneration

**Fig. 5.**
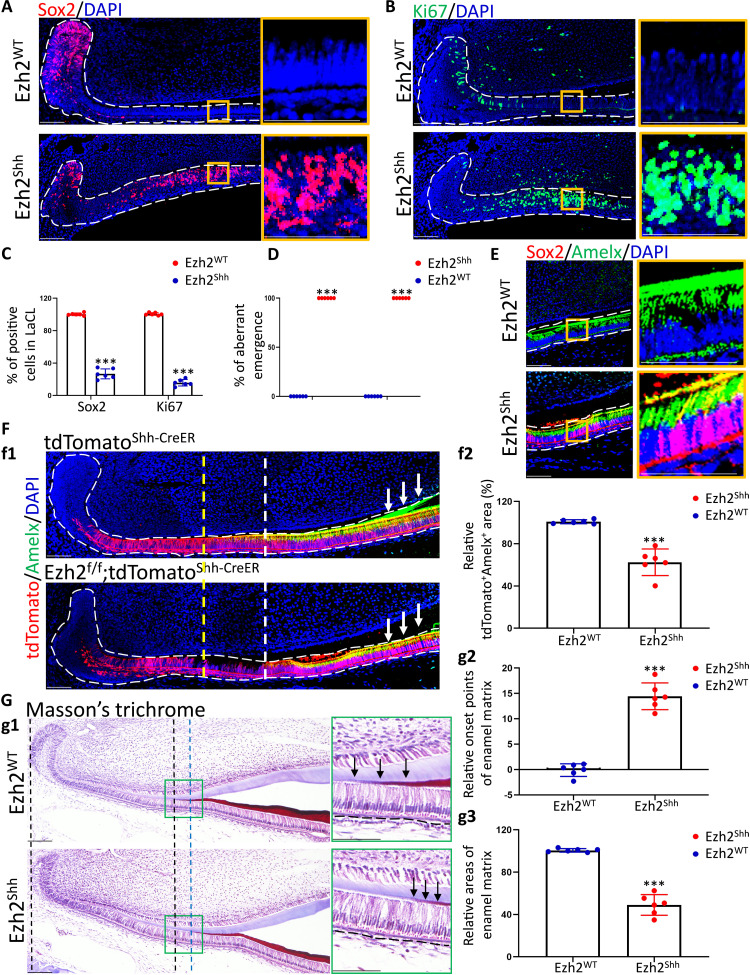
Knocking out of Ezh2 caused lineage infidelity of incisor epithelium and thus impaired enamel matrix production capability during regeneration. (**A**) Immunofluorescent images of Sox2 at d5 after clipping. (**B**) Immunofluorescent images of Ki67 at d5 after clipping. (**C**) Statistical analysis on the number of Sox2^+^ or Ki67^+^ cells in LaCL region of (A) and (B). (**D**) Statistical analysis on the ratio of aberrant emergence in (A) and (B). (**E**) Double-staining immunofluorescent images of Sox2 and Amelx in secretory ameloblast region at d5 after clipping. (**F**) f1: Double-staining immunofluorescent images of tdTomato and Amelx at d5 after clipping. f2: Statistical analysis on tdTomato^+^Amelx^+^ area in f1. (**G**) g1: Masson’s trichrome images at d5 after clipping. The arrows in the high magnification indicated enamel matrix, which was dyed red. g2: Statistical analysis on the location of the onset points of enamel matrix in g1. g3: Statistical analysis on the area of enamel matrix in g1. All representative images and data (means ± SEM) were generated from at least six slices from six mice for each group. ***P < 0.001. The dot-square contoured regions were shown in the right as high-magnification images. Scale bar, 50 μm.

On the basis of our homeostatic results, we hypothesized that the disruption of cell fate in Ezh2^Shh^ may also occur during regeneration, by which impaired enamel regeneration was caused. To verify this, we carried out the following experiments. Consequently, data showed that both Sox2^+^ and Ki67^+^ cells were reduced in LaCL and moved forward to the ameloblast region in the Ezh2^Shh^ incisor at d5 after injury (Fig. 5, A to D). Some high columnar cells in the Ezh2^Shh^ incisor were Sox2^+^ or Ki67^+^, which would not occur in WT incisors (Fig. 5, A and B). Further double staining showed that these Sox2^+^ high columnar cells were also Amelx^+^, suggesting loss of stemness of Sox2^+^ population (Fig. 5E). However, unlike that on intact condition, the onset of Amelx expression moved distally to LaCL in Ezh2^Shh^ incisor at d5 after injury (Figs. 4B and 5F). Additional double staining of tdTomato and Amelx demonstrated reduced Amelx secretion of Shh^+^ lineage, suggesting the loss of amelogenesis capability after Ezh2 depletion (Fig. 5F). To confirm this, we did MTS and observed delayed and decreased enamel matrix deposition, which were consistent with what we observed in μCT (Fig. 5G). Thus, experiments above revealed that Ezh2 depletion disrupted cell fate and impaired epithelium-derived enamel regeneration.

To tease out the effects of long-term Ezh2 KO on Shh expression and stem cell exhaustion, we changed administration strategy via injecting EED226 at d1 after clipping as described above (fig. S2C). Following this strategy, our data clearly demonstrated that timely inhibition of Ezh2/H3K27me3 signaling at 1 day after clipping led to the failure of pulling injury-induced Shh back to homeostatic level; therefore, it has proved the repressive effect of Ezh2/H3K27me3 on Shh in injury, too (fig. S2G). IF data of Sox2 furthermore showed that at d2 after clipping following this strategy, there was no significant alteration of Sox2^+^ cells in EED226 and dimethyl sulfoxide groups (fig. S2H). However, at d3 and d5 after clipping, data showed that along with the accumulation of Ezh2/H3K27me3 inhibition, Sox2^+^ populations began to decline (fig. S2H). This may explain the reasons for stemness exhaustion that occurred in long-term Ezh2 KO.

### Ezh2/H3K27me3 upstream controlled Shh to safeguard the fidelity of ASC fate decision

Soon after incisor clipping, injury-induced Shh expressions in IEE and PAB regions were observed, and the significantly increased H3K27me3 was also observed in the same regions (Fig. 1, E to G). However, when it came to homeostasis, this injury-induced Shh was pulled back to low extents; therefore, injury-induced epigenetic signal of Ezh2/H3K27me3 was hypothesized to be the key of pulling Shh expression back to homeostatic level (Fig. 1, E to H). In adult murine schwann cells, Shh has been proved to be highly H3K27me3-occupied, and H3K27me3 did directly restrain Shh in injury (*15*). Therefore, next, we sought to ascertain whether epithelial Shh was upstream regulated by Ezh2/H3K27me3 in DESCs and whether by such mechanism cell fate determination was orchestrated.

**Fig. 6.**
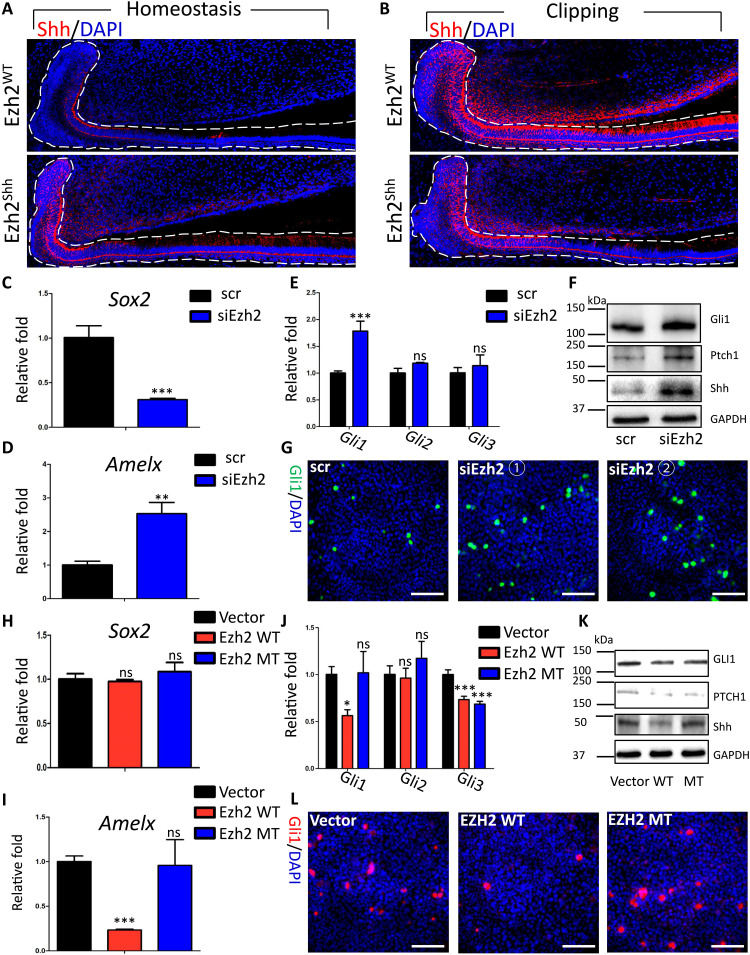
Ezh2/H3K27me3 upstream controlled Shh to safeguard the fidelity of ASC fate decision. (**A** and **B**) Immunofluorescent image of Shh on intact condition or at d5 after injury. (**C** to **E** and **H** to **J**) RT-qPCR data of *Sox2*, *Amelx*, and *Gli1-3*. (**F** and **K**) Western blot images of Shh/Ptch1/Gli1 signaling. (**G** and **L**) Immunofluorescent image of Gli1 in DESCs cultured in vitro with Ezh2 knockdown or overexpression, respectively. In vivo representative images were generated from at least six slices from six mice for each group. Experiments in DESCs were repeated at least three times with three duplicates for each group. Data are means ± SEM; *n* = 3. *P < 0.05, **P < 0.01, and ***P < 0.001. Scale bar, 50 μm. GAPDH, glyceraldehyde-3-phosphate dehydrogenase.

Our in vivo data showed that after depleting Ezh2 in Shh^+^ cells, the expressions of Shh were significantly up-regulated, indicating the repressive function of Ezh2/H3K27me3 on epithelial Shh (Fig. 6A). However, at d5 after clipping, epithelial Shh of Ezh2^shh^ incisors failed to be induced by injury, suggesting that long-term disrupted cell fate caused significantly impaired regenerative responses (Fig. 6B). Next, we cultured DESCs in vitro to ultimately make sure whether Ezh2/H3K27me3 was the direct upstream inhibitor of Shh and to ascertain if such epigenetic control of Shh determined cell fate. Isolation of LaCL and culture of DESCs were performed according to a method reported previously (*27*). Data of DESC identification are shown in fig. S1 (C to J). Consequently, our in vitro data demonstrated that silencing Ezh2 in DESCs led to obviously reduced Sox2 but increased Amelx, implying impairment of stemness and initiation of differentiation (Fig. 6, C and D). Meanwhile, we observed up-regulated Shh and its downstream Ptch1 and Gli1 in Ezh2-silenced DESCs, indicating the activation of Shh signaling (Fig. 6F). We furthermore found that it was Gli1, not Gli2 or Gli3, that responded to Ezh2 silence (Fig. 6E). IF data of nuclear Gli1 furthermore proved the activation of Shh/Gli1 in Ezh2-difficient DESCs (Fig. 6G). Next, we detected whether these effects of Ezh2 in DESCs were H3K27me3 methylation dependent. Although we did not observe increased Sox2 after overexpressing Ezh2 in DESCs, reduced Amelx occurred. The repressive effect of Ezh2 overexpression on Amelx level was abolished after deleting the H3K27me3 methylation domain (Ezh2 MT) (Fig. 6, H and I). Together with small interfering RNA (siRNA) data, we concluded that the functions of Ezh2/H3K27me3 in DESCs were to maintain stemness and restrain differentiation. Next, the repressive effects of Ezh2 overexpression on Shh/Ptch1/Gli1 signaling in DESCs were also confirmed to be H3K27me3 methylation dependent (Fig. 6K). Besides, it was mutually corroborated by Ezh2 silence and overexpression experiments that it was Gli1 rather than Gli2 or Gli3 that was the downstream of Ezh2/H3K27me3 effector (Fig. 6J). Last, nuclear Gli1 detection testified that overexpressing Ezh2 repressed Shh/Gli1 signaling in DESCs, but the absence of H3K27 methylation domain abolished such function (Fig. 6L).

### Ezh2-controlled Shh expression was H3K27 trimethylation dependent

**Fig. 7.**
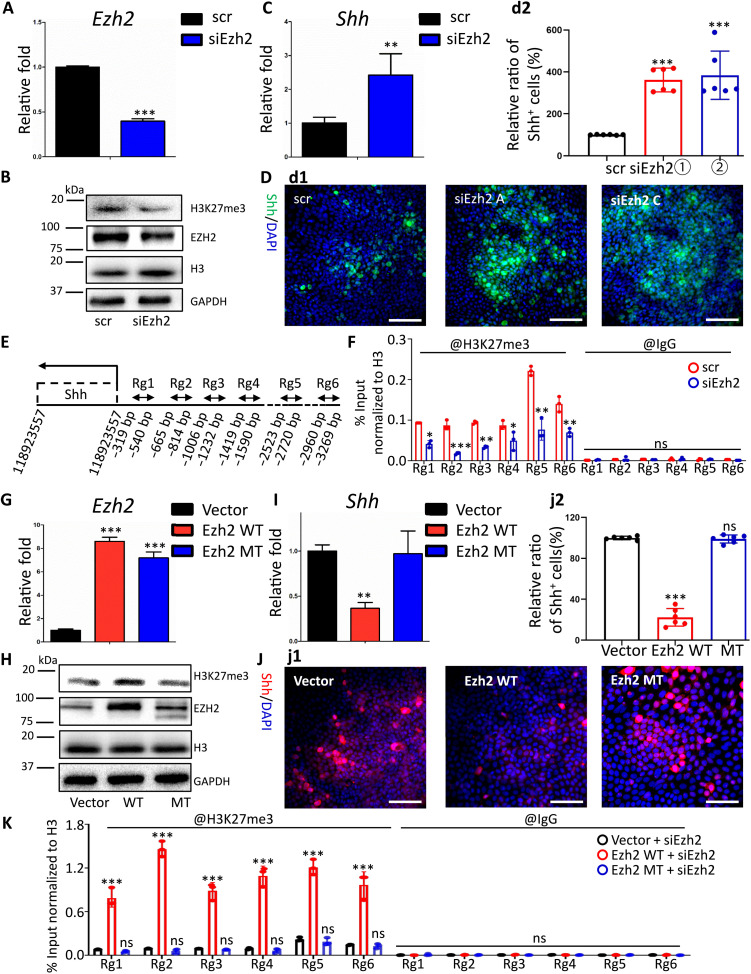
Ezh2-controlled Shh expression was H3K27 trimethylation dependent. (**A**, **C**, **G**, and **I**) RT-qPCR data of *Ezh2* and *Shh*. (**B** and **H**) Western blot images of Ezh2 and H3K27me3. (**D** and **J**) d1 and j1: Immunofluorescent image of Shh. d2 and j2: Quantification of Shh^+^ cell in d1 and j1. (**E**, **F**, and **K**) ChIP-qPCR data of H3K27me3 within the TSS (−3000 to 0 bp) region of Shh gene. Experiments in DESCs were repeated at least three times with three duplicates for each group. Data are means ± SEM; *n* = 3. *P < 0.05, **P < 0.01, and ***P < 0.001. Scale bar, 50 μm.

To investigate the molecular mechanism by which Ezh2 regulates Shh level, we separately silenced or overexpressed Ezh2 in DESCs. First, via knocking down Ezh2 through specific siRNAs, we detected weakened H3K27me3 modification and increased Shh both at mRNA and protein levels (Fig. 7, A to D). This was further corroborated by chromatin immunoprecipitation (ChIP), which pulled down more H3K27me3-modified DNA fragments within the transcription start site (TSS) [about −3000 to 0 base pair (bp)] of Shh gene from control DESCs than that from Ezh2-knockdown samples, confirming that Ezh2 directly regulated Shh expression via catalyzing H3K27me3 (Fig. 7, E and F).

As a core enzymatic component of PRC2 complex, Ezh2 contains two functionally different domains, namely, WD-binding domain and SET domain (*28*–*30*). The WD-binding domain binds to WD-repeat domain of EED and thus facilitates PRC2 formation, while the SET domain performs histone methyltransferase activity (*29*–*30*). To investigate whether Ezh2 regulates Shh expression in a methyltransferase activity–dependent manner, wild-type Ezh2 (Ezh2 WT) and SET domain–deleted mutant (Ezh2 MT) Ezh2 were respectively overexpressed in DESCs. Our data showed that overexpression of Ezh2 WT, instead of Ezh2 MT, enhanced H3K27me3 modification and accordingly decreased Shh expression both at mRNA and protein levels (Fig. 7, G to J). ChIP assay further confirmed that Ezh2-controlled Shh expression was H3K27 trimethyltransferase activity dependent. In detail, overexpressing Ezh2 WT significantly increased H3K27me3 modification within TSS of Shh, while Ezh2 MT failed to increase H3K27me3 occupation (Fig. 7K). To confirm that the enrichment changes of H3K27me3 within the Shh promoter were reliable, we set Sox2 gene as a control that showed no H3K27me3 occupation alterations both in silence and overexpression conditions (fig. S3).

## DISCUSSION

In this study, we revealed that Ezh2-mediated H3K27me3 upstream repressed Shh expression to orchestrate incisor epithelial ASC fate, thus maintaining tissue homeostasis and regeneration. In homeostatic epithelium, highly preimprinted H3K27me3 in OEE/stellate reticulum (SR) of LaCL and ameloblasts contoured the space-wise expression of Shh, which was only present within TA and PAB zones with decreased H3K27me3. Accordingly, it can be assumed that the unique spatial pattern of H3K27me3 is to precisely limit the expression scope of Shh. Considering the essential role of Shh for DESC proliferation and amelogenic differentiation (*9*–*11*), we supposed that the space-wise expression of Shh sustained mitotic progenitors in TA and PAB zones and avoided disruptions of quiescent stem cells in LaCL. This hypothesis was verified by our genetic cKO data of depleting Ezh2 in Shh^+^ lineage, which showed severely disrupted cell fate in homeostatic incisor epithelium after erasing H3K27me3. In detail, loss of repressive functions of H3K27me3 increased Shh expressions in TA and PAB, which, in turn, contributed to excessive Ki67^+^ proliferating cells, aberrantly cycling Sox2^+^ cells and premature Sox2^+^ ameloblasts. These findings clearly demonstrated that the fidelity of cell fate determination was indispensable for adult tissue homeostasis, and specifically, Ezh2/H3K27me3-controlled Shh transcription was in charge of incisor ASC fate decision.

In addition to homeostasis, Ezh2/H3K27me3-mediated Shh repression also regulated ASC-based enamel regeneration. During regeneration of incisors, we observed dynamic alterations of cell fate in epithelium, including the recruitment of Sox2^+^ DESCs, increase of Ki67^+^ cycling progenitors, and delayed onset of Amelx^+^ ameloblasts. According to previous reports, together, these changes were to provide more cell resources required for regeneration (*5*). As for the upstream regulators, injury-induced Shh was supposed to facilitate these cell fate alterations, because Shh has been proved to regulate proliferation and differentiation of DESCs (*9*–*13*), and synchronous increment of Shh and extended Sox2^+^/Ki67^+^ regions were observed after depleting Ezh2 in our study. Although Shh was proposed to facilitate enamel regeneration (*9*), our data revealed that injury-induced Shh must be precisely restrained so that to subsequently rebuild tissue homeostasis. In detail, long-term elevation of Shh after Ezh2 depletion significantly disordered epithelial cell fates, including reduction of Sox2^+^ quiescent DESCs in LaCL, appearance of aberrantly cycling Sox2^+^ cells, and premature Sox2^+^ ameloblasts in TA and PAB, respectively. These cumulative cell fate disruptions resulted in stem cell exhaustion in Ezh2^shh^ epithelium; as a result, both Shh expression and amelogenesis could not be supported after injury, therefore failing to regenerate enamel. According to these data, increment of Ezh2/H3K27me3 after injury was supposed to pull injury-induced Shh to homeostatic levels, thus rebuilding homeostasis and maintaining regeneration capability. Together, the repressive function of Ezh2/H3K27me3 on Shh guaranteed both homeostasis and regeneration of rodent incisors. Despite achievements mentioned above, several issues remain to be investigated in the future. In detail, it is still unclear about the upstream signals that raise Ezh2 and Shh soon after injury and whether there exists a more primitive quiescent stem cell population residing within OEE and SR that generates Sox2^+^ DESCs responding to injury signals (*25*).

As our in vivo and in vitro data of deleting Ezh2 showed up-regulation of Shh, we concluded the repressive role of Ezh2 in Shh expression of incisor epithelium. Intriguingly, we observed that long-term loss of Ezh2 had led to severely disrupted cell fate determination in intact incisor epithelium. Thus, when severe injury happened, stemness exhaustion emerged to insufficiently generate Shh-expressing progenies, so that long-term cKO of Ezh2 in epithelium made it fail to meet the requirements of vast stemness and rigor lineage commitment after clipping, resulting in impairment of regeneration. Specifically, after long-term loss of Ezh2, clipping led to severely disrupted cell fates in injured epithelium, which exhibited as follows: (i) Quiescent Sox2^+^ DESCs and Ki67^+^ progenitors in LaCL markedly decreased; (ii) the Sox2^+^ cells aberrantly emerged at ameloblast zones, which apparently had lost stemness characteristics as they became Ki67^+^ actively proliferating and Amelx^+^Sox2^+^ prematurely differentiated cells; and (iii) much delayed amelogenic differentiation and decreased secretion of enamel matrix. Under clipping conditions, these accumulated disorders consequently caused irreversible cell fate chaos and stemness exhaustion, thus unable to supply the demand for stemness and accurate lineage commitment during regeneration. As a consequence, barely normal and functional TA and PAB cells, the Shh-expressing progenies of DESCs, were generated, leading to declined Shh in injured Ezh2 cKO epithelium.

Fidelity of ASC fate determination is crucial for tissue homeostasis and regeneration (*1*–*4*). It is largely unknown whether epigenetic signals, as reversible regulatory machinery, indispensably participate in this fidelity control of adult teeth. Here, we forward prove that H3K27 methylation writer Ezh2 indispensably regulates the fidelity of incisor epithelial ASC fate determination via restraining Shh expression. The dynamic alterations of Ezh2/H3K27me3 in injury guarantee epithelium to meet the vast stemness needs and precise descendant generation for regeneration. Furthermore, this epigenetic machinery helps rebuild homeostatic condition when regeneration was accomplished. This finding exactly shows the important characteristics of epigenetic regulations for controlling organohomeostasis and regeneration, which are irreversible, dynamic, and signal-amplified. It deserves more investigations to mechanistically reveal the coordination between epigenetic writers, readers, and erasers in organ homeostasis and regeneration; theoretically, these three basic epigenetic elements can compose a very subtle and comprehensive signal hub.

To sum up, our study demonstrated the very importance of ASC fate determination for maintaining tissue homeostasis and regeneration. In detail, we uncovered that the spatiotemporal dynamics of Ezh2/H3K27me3 between homeostasis and regeneration controlled ASC fate and tissue regeneration, and the underlying mechanisms were H3K27me3-mediated Shh repression. Discoveries in this research will help us better understand the role of ASC fate determination in tissue homeostasis and regeneration and endow researchers with potential knowledge to ignite ASC-mediated tissue regeneration.

## MATERIALS AND METHODS

### Ethics statement and animal strains

This study was approved by the Ethics Committees of West China School of Stomatology, Sichuan University (approval number WCHSIRB-D-2017-024). All animal experiments strictly complied with the ethical requirements. Shh-CreER (Stock #005623), tdTomato (Stock #007914), and Ezh2^fl/fl^ (Stock #022616) strains were purchased from the Jackson laboratory. In addition, C57/B6J mice were purchased from Chengdu Dashuo Experimental Animals Company (China). All animal studies conformed to ARRIVE (Animal Research: Reporting of In Vivo Experiments) guidelines. Briefly, all mice were housed in specific pathogen–free Experimental Animal Core of West China Hospital, Sichuan University (China), which was a temperature-controlled (25°C) environment under a 12-hour light/12-hour dark cycle with cotton batting. To generate Shh-CreER; tdTomato strain, we crossed Shh-CreER with tdTomato, and to get Shh-CreER; Ezh2^fl/fl^ strain, we crossed Shh-CreER with Ezh2^fl/fl^. We followed the tamoxifen (TAM) dosing in our previous publication (70 mg TAM/kg weight, interperitoneally), which showed the efficacy and safety of this dosing in analyzing hard tissues (*31*).

### Experimental model and subject details

Detailed information of materials and oligo primers are shown in the Supplementary Materials.

### Injury-induced incisor regeneration model

Mandibular incisors of 3-month-old mice were clipped at the gingival level, making sure that dental pulp was exposed (fig. S1A). The distance between the incisal edge of the intact side to the defect edge of the injury side was defined as defect length. These mice were then followed for 5 days to observe regeneration of injured incisors.

### Isolation and culture of DESCs

We isolated LaCL, the putative nich of incisor epithelium, according to a method reported before (*27*). This LaCL tissue was then subjected to culture of DESCs using a verified in vitro culture system. The details are as follows: Mandibular incisors of 3-month-old mice were dissected with #15 scapel and placed in cold phosphate-buffered saline (PBS) immediately. Bone around the incisors was removed under a microscope using micro tweezers. Incisors were then placed in 2% collagenase type I in PBS for 2 hours at 4°C in a six-well plate. The mesenchyme closed to dental epithelium was gently separated using syringes (0.5 ml, 28G1/2). Then, the labial epithelium that surrounds apical papilla but extends no more than dentin matrix was microdissected as LaCL. These LaCL tissues were immediately placed in cold Dulbecco’s modified Eagle’s medium (DMEM)/F12 in a 1.5-ml centrifuge tube. Then cell culture of DESCs was performed according to the protocol described previously. The cells were maintained in DESC media, consisting of DMEM/F12 supplemented with mouse epidermal growth factor recombinant protein at a concentration of 20 ng/ml, fibroblast growth factor recombinant protein at a concentration of 25 ng/ml, 1× B27 supplement, and 1% antibiotic solution (penicillin, 100 U/ml; streptomycin, 50 mg/ml). DESCs from passage 1 were used for experiments.

### ChIP analysis

Chromatin from cultured cells was prepared using the EZ-Zyme Chromatin Prep Kit. Immunoprecipitation of cross-linked protein/DNA was performed with the EZ-Magna ChIP HiSens Chromatin Immunoprecipitation Kit. Both chromatin preparation and ChIP assay were performed exactly following the protocols supplied by manufacturers. The purified DNA was analyzed by quantitative real-time PCR. The primers used for ChIP-qPCR assay are listed in table S1. All ChIP-qPCR data were normalized to H3 enrichment. For overexpression experiments, the endogenous Ezh2 were presilenced at 48 hours before overexpression viruses infected.

### Immunostaining

For IF staining of paraffin sections, mandibular incisors of 3-month-old mice were dissected with #15 scapel. Samples were fixed in 4% paraformaldehyde overnight at 4°C, followed by decalcification in 10% EDTA for 4 weeks and dehydration through serial concentration of ethanol for embedding in paraffin. Samples 4 μm thick were prepared using Ultra-Thin Microtome (Leica RM2235). The slides were then heated in a 60°C oven for 24 hours, followed by dewaxing and hydration through xylene and a series of decreasing concentrations of ethanol. After preheating the antigen-unmasking solution in a microwave oven to a temperature of 95° to 100°C, deparaffinized samples were immersed in antigen unmasking solution and incubated at 99°C for 30 min, followed by cooling down at room temperature for 30 min. Next, samples were permeabilized with 0.5% Triton X-100 in PBS for 10 min and blocked by bovine serum albumin (BSA; 0.01 g/ml) for 30 min at room temperature. For immunocytofluorescence staining, DESCs of P1 were seeded in 48-well plates to form colonies. Then, these cells were transfected with siRNA or adenovirus. Forty-eight hours after transfection, cell samples were fixed in 4% paraformaldehyde for 15 min at room temperature, followed by permeabilization with 0.5% Triton-X in PBS for 10 min and BSA blocking (0.01 g/ml) for 30 min at room temperature. After blocking, samples were incubated overnight in primary antibodies with appropriate concentration at 4°C. Then, samples were washed by PBS for 15 min three times. After PBS washing, secondary antibodies were used to incubate these samples for 60 min at room temperature. The cell nuclei were also labeled with 4′,6-diamidino-2-phenylindole.

For quantification of immunocytofluorescence, we used Image-Pro Plus 7.0 (Media Cybernetics, MD, USA). Briefly, the measurement tool of Image-Pro Plus 7.0 was used to select and classify fluorescence-positive objects by color and locate and segment objects on the basis of their color. Then, we created classes to further characterize and streamline data collection and reporting.

### μCT assessment

Incisors, isolated from euthanized mice, were fixed in 4% paraformaldehyde at 4°C overnight and transferred to PBS for storage. The Scanco Medical μCT 35 System (Scanco Medical AG, Bassersdorf, Switzerland) was used for following scan. Parameters were selected according to a previously established program: x-ray intensity, 145 μA; integration time, 200 ms; x-ray tube potential, 55 kVp; threshold, 220 mg/cm^3^.

### Ezh2 interference with siRNAs or adenovirus

DESCs were transfected with Ezh2-specific siRNAs at a concentration of 30 nM via Lipofectamine 3000 reagent according to protocols provided. Adenoviruses that that contain full-length WT Ezh2 or SET domain–deleted Ezh2 (Ezh2 ΔSET) or vectors were purchased from HANBIO Technology Co. Ltd., Shanghai. DESCs were infected with adenoviruses for 8 hours, followed by change of fresh media. Cells were harvested 24 or 48 hours later after transfection. RT-qPCR and Western blotting were used to determine the efficiency of Ezh2 abrogation or overexpression.

### Quantitative real-time PCR analysis

Total RNA from incisor epithelium tissues or cultured DESCs was extracted using the RNeasy Micro Kit according to the manufacturer’s protocol. Complementary DNA (cDNA) was synthesized by using HiScript III RT SuperMix for a qPCR kit. Above, cDNA was subsequently used in the AceQ Universal SYBR qPCR Master Mix Kit for qPCR reactions. Glyceraldehyde-3-phosphate dehydrogenase (GAPDH) was selected as a control. The primer sequences and conditions for real-time PCR are listed in table S1. A single melting peak was obtained for all qPCR products. The results were calculated applying the ΔΔCT method and presented as fold changes relative to GAPDH.

### Western blotting

Cells were washed three times with PBS and incubated on ice for 15 min in M-PER Mammalian Protein Extraction Reagent containing Halt Protease and Phosphatase Inhibitor Cocktail. The total cell protein was extracted following the manufacturer’s instructions. Twenty-microgram protein for each sample was loaded on a 10% polyacrylamide gel and then transferred to the polyvinylidene difluoride (PVDF) membrane. After being blocked for 1 hour with BSA (0.05 g/ml) in triethanolamine-buffered saline containing 0.5% Tween 20 (TBST), the PVDF membranes were incubated at 4°C with primary antibodies of appropriate concentrations. After overnight incubation, PVDF membranes were washed by TBST for 5 min three times, followed by a 1-hour incubation with horseradish peroxidase (HRP)–conjugated immunoglobulin G antibodies. Next, PVDF membranes were washed again using TBST for 5 min three times. Proteins bands were visualized using Luminata Forte Western HRP substrate. ImageJ software was used to conduct quantitative analysis.

### Colony formation assay

Single-cell suspension of DESCs (1 × 10^4^) was seeded into six-well culture plates. After 14 days, the culture plates were fixed in 4% paraformaldehyde for 15 min at room temperature. After fixation, DESCs were stained with 0.1% crystal violet. Colonies containing >50 cells were counted as single-colony clusters.

### Histological staining

H&E and MTS staining were performed using a commercial staining kit (table S1) in accordance to kit protocol.

### Statistical analysis

For each experiment, we have at least three independent biological repeats. A two-tailed Student’s *t* test was applied for statistical analysis. A *P* value of <0.05 was considered statistically significant.
