# Supplementary material for: Epigenetic controls of Sonic hedgehog guarantee fidelity of epithelial adult stem cells trajectory in regeneration
Source: Sci Adv. 2022 Jul 22;8(29):eabn4977. doi: 10.1126/sciadv.abn4977 (PMC9307244; doi:10.1126/sciadv.abn4977)
Supplement: Supplementary file 1 — Figs. S1 to S3 Table S1 [file sciadv.abn4977_sm.pdf]

Science Advances
AAAS

Supplementary Materials for

Epigenetic controls of Sonic hedgehog guarantee fidelity of epithelial adult stem cells trajectory in regeneration

Fanyuan Yu *et al.*

Corresponding author: Liwei Zheng, liwei.zheng@scu.edu.cn; Ling Ye, yeling@scu.edu.cn

Sci. Adv. **8**, eabn4977 (2022)
DOI: 10.1126/sciadv.abn4977

This PDF file includes:

Figs. S1 to S3
Table S1

Supplemental Figure Legends

Fig S1

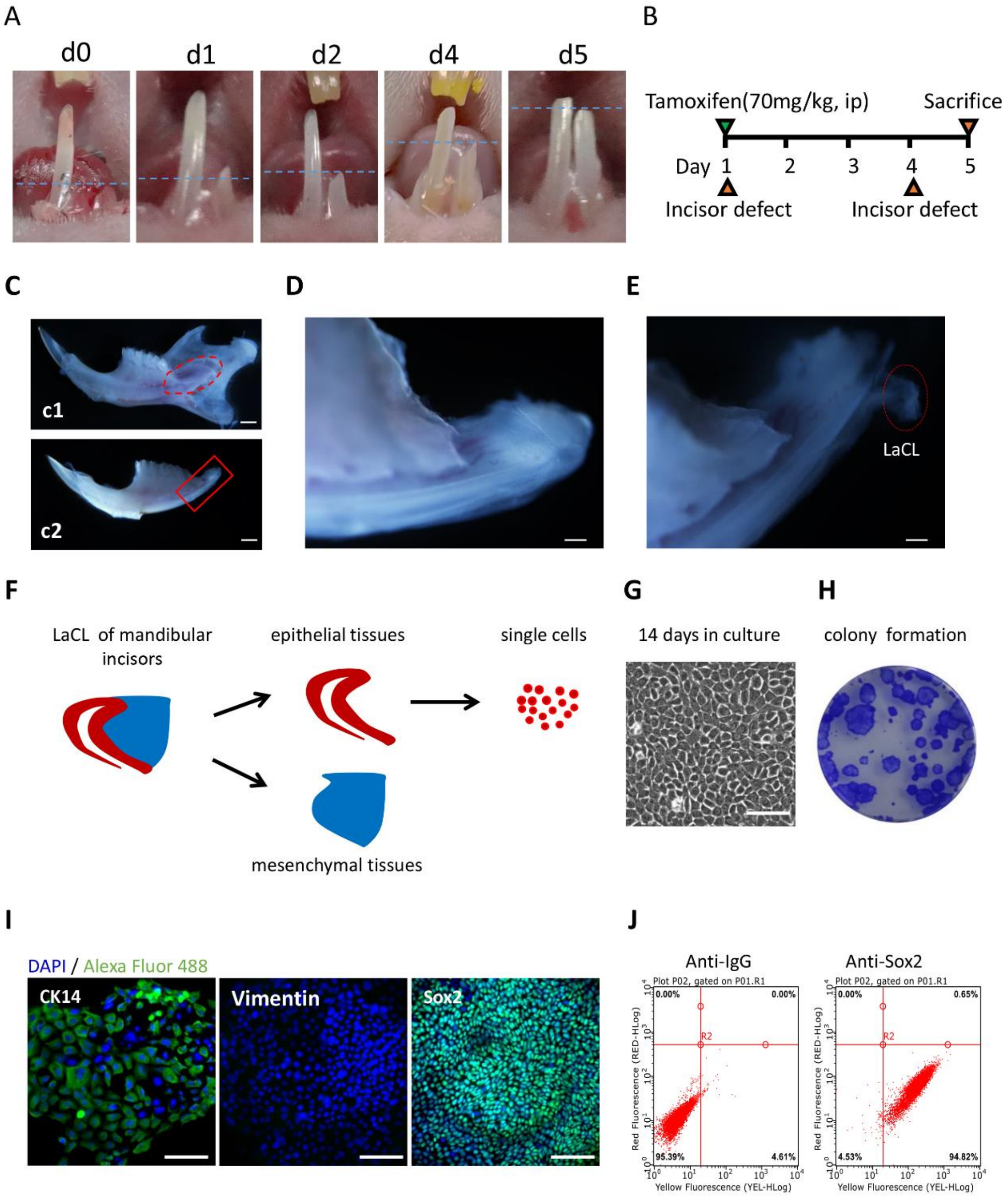

Supplemental figure 1. (A) Photograph images of incisors at different time points after clipping. (B) Schematic illustration of tamoxifen administration plan and the time points selected to clip incisors in Fig. 2I; ip, interperitoneal injection. (C-E) Stereomicroscopic images of dissection and isolation of LaCL: (c1-c2) mandibular incisors before or after surrounding jaw bone was removed; (D) high magnification of apical bud that was boxed in c2; (E) LaCL that was mechanically isolated. (F) Schematic images depicting isolation and culture of DESCs. (G) Microscopic image in

bright field. (H) Crystal violet staining 14 days after primary cell culture. (I) Immunofluorescent images of CK14, Sox2 and Vimentin in DESCs cultured *in vitro*. (J) Cytoflow analysis on Sox2 positive percentages in primary DESCs cultured *in vitro*. In vivo representative images were generated from at least six slices from six mice for each group. Experiments in DESCs was repeated at least three times with three duplicates for each group. Scale bars, 50 μm in C, D and E; 25 μm in G and I.

Fig S2

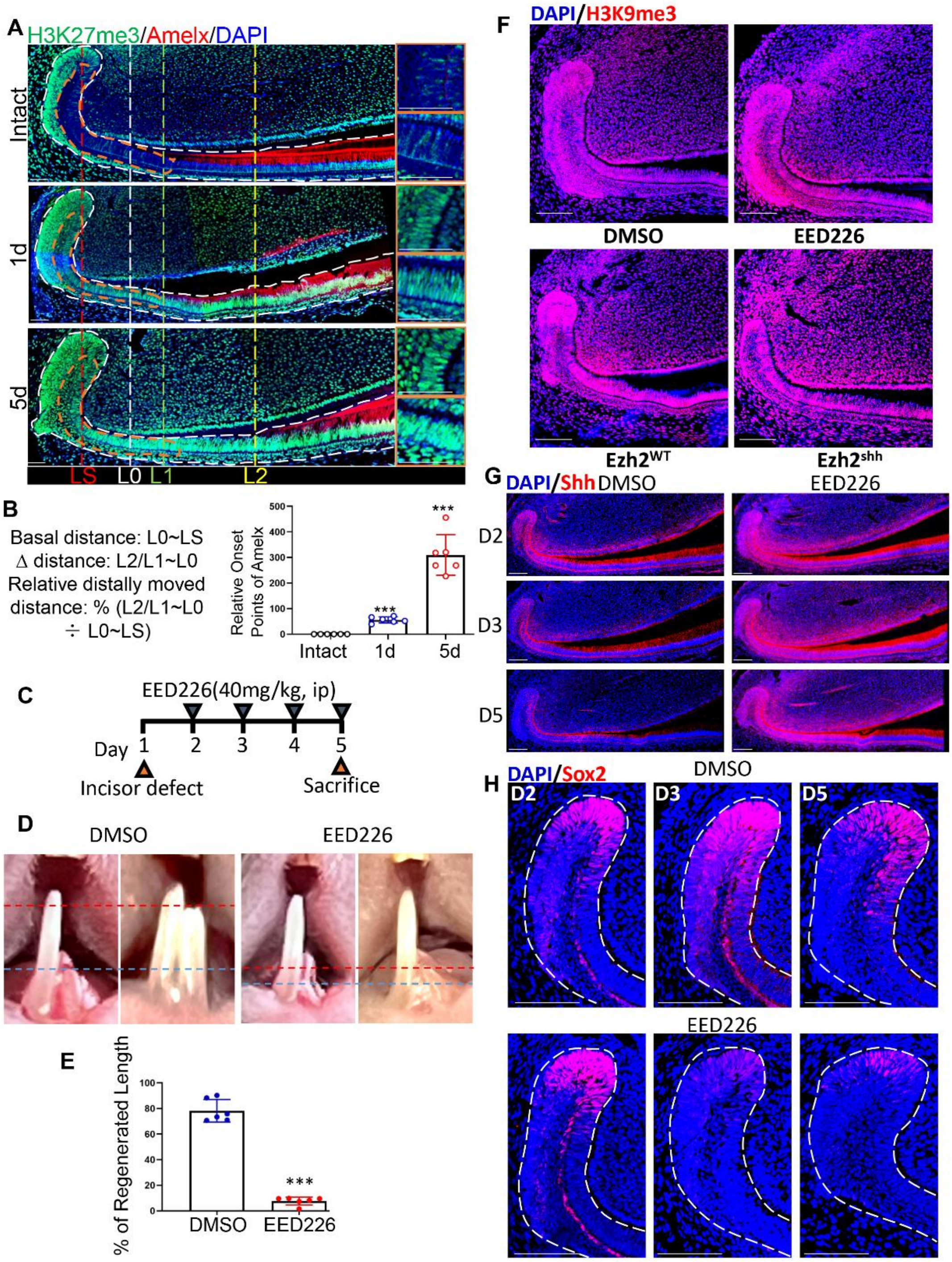

Supplemental figure 2. (A) Double staining immunofluorescent images of H3K27me3 and Amelx. LS, line showing the summit of LaCL; L0, line showing start site of Amelx

deposit in intact condition; L1, line showing start site of Amelx deposit at 1d-post clipping; L2, line showing start site of Amelx deposit at 5d-post clipping. Injury-induced-H3K27me3 in IEE at day1 and 5 post-clipping were dotted in brown. (B) The relative distally moved distance about the onset point of Amelx expression in (A). (C) Timeline schematic of the EED226 administration strategy which were used in (D), (E), (G), and (H). (D) Photographs of incisor at d5-post clipping. (E) Statistic data of relative regenerated length at d5-post clipping. (F) Representative IF images of H3K9me3 in intact incisors. (G) Representative IF images of Shh. (H) Representative IF images of Sox2. ***, p<0.001. Scale bar, 50 µm.

Fig S3

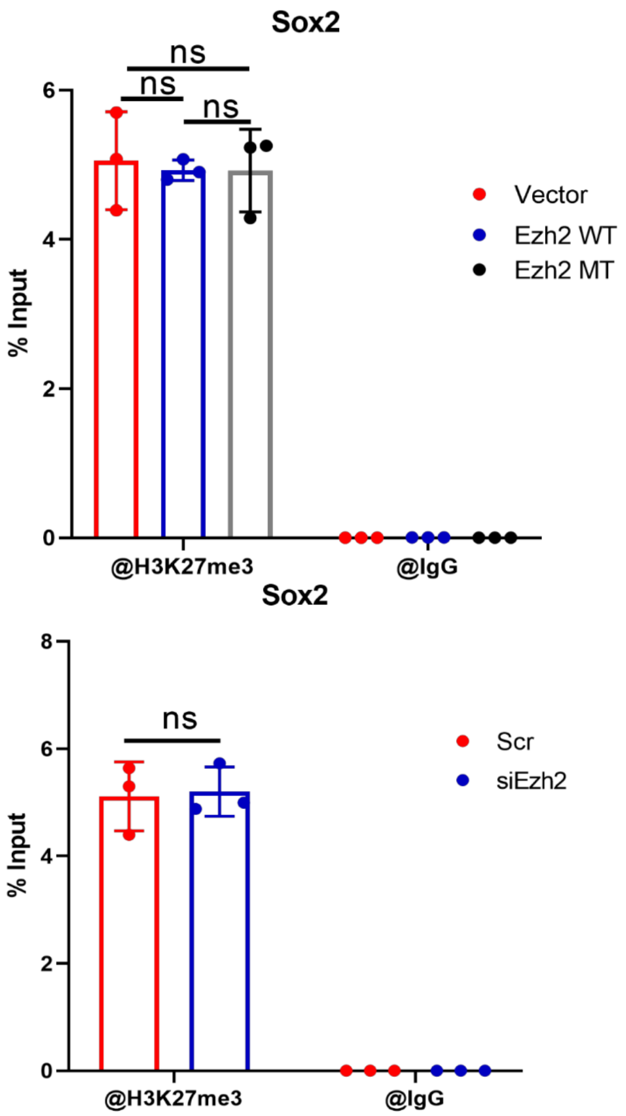

Supplemental figure 3. ChIP-qPCR data of H3K27me3 within the promoter region of Sox2 gene. Experiments in DESCs was repeated at least three times with three duplicates for each group. Data are means ± SEM; n = 3. ns, no statistical significance.

Supplemental Table S1

Key Resources Table

Reagent or Resource	Source	Identifier

Antibodies		
Anti-Tri-Methyl-Histone H3 (Lys27) (H3K27me3) Rabbit mAb	Cell Signaling	Cat#9733
Anti-Tri-Methyl-Histone H3 (Lys9) (H3K9me3) Rabbit mAb	Cell Signaling	Cat#13969
Anti-Ezh2 Rabbit mAb	Cell Signaling	Cat#5246
Anti-Sox2 Rabbit mAb	Abcam	Cat#ab92494
Anti-Ki67 Rabbit mAb	Abcam	Cat#ab16667
Anti-Shh Rabbit mAb	Abcam	Cat#ab53281
Anti-mCherry Rabbit mAb	Abcam	Cat#ab167453
Anti-Amelogenin Mouse mAb	Santa Cruz	Cat#sc365284
Anti- Keratin 14 Rabbit mAb	Abcam	Cat#ab181595
Anti- Vimentin Rabbit mAb	Abcam	Cat#ab92547
Anti-Ptch1 Rabbit polyclonal Ab	SAB	Cat #35897
Anti-Gli1 Rabbit polyclonal Ab	SAB	Cat#43926
Anti-Histone H3 Rabbit mAb	Cell Signaling	Cat# 60932
Anti-Shh Rabbit polyclonal Ab	Novus	Cat# NBP2-22139
Normal Rabbit IgG	Cell Signaling	Cat# 2729
Goat anti-Rabbit IgG-H&L (Alexa Fluor® 647)	Abcam	Cat# ab150087
Goat anti-Rabbit IgG-H&L (Alexa Fluor® 488)	Abcam	Cat# ab150077
Goat anti-Mouse IgG-H&L (Alexa Fluor® 488)	Abcam	Cat# ab150117
Goat anti-rabbit IgG (HRP)	Abcam	Cat# ab6721
Goat anti-mouse IgG (HRP)	Abcam	Cat# ab6789
Bacterial and Virus Strains		
Ezh2 WT	Hanbio	In this paper
Ezh2 MT	Hanbio	In this paper
Ezh2 vector (mCherry)	Hanbio	In this paper
Chemicals, and Recombinant Proteins		
EED226	Selleck	Cat#S8496
Dulbecco's Modified Eagle Medium/Nutrient Mixture F-12 (DMEM/F12)	Gibco	Cat# 11320033
B-27 Plus Supplement	Gibco	Cat# A3582801
Penicillin-streptomycin (P/S)	Gibco	Cat#15140122
Recombinant Mouse FGF basic Protein	R&D Systems	Cat#3139-FB-025/CF
Recombinant Mouse EGF Protein	R&D Systems	Cat#2028-EG-200
4% paraformaldehyde	Boster	Cat#AR1068
HiScript III RT SuperMix for qPCR	Vazyme	Cat#R323-01
AceQ Universal SYBR qPCR Master Mix	Vazyme	Cat#Q511-02

Triton X-100	Thermo Scientific	Cat#85111
PBS	Boster	Cat#AR1155
Dimethyl sulfoxide (DMSO)	Sigma-Aldrich	Cat#D8418
DAPI Staining Solution	Beyotime	Cat#C1005
Tamoxifen	Sigma-Aldrich	Cat#T5648
Lipofectamine™ 3000 Transfection Reagent	Invitrogen	Cat#L3000001
Halt™ Protease and Phosphatase Inhibitor Cocktail	Thermo Scientific	Cat#78441B
Luminata Forte Western HRP substrate	Millipore Corp	Cat#WBLUC0100
Experimental Models: Mouse Lines		
B6.129S6-Shhtm2(cre/ERT2)Cjt/J	JAX Lab	Stock#005623
tdTomato	JAX Lab	Stock#007914
B6;129S1-Ezh2tm2Sho/J	JAX Lab	Stock# 022616
C57/B6J	Chengdu Dashuo Experimental Animals Company	C57/B6J
Critical Commercial Assays		
HiScript III RT SuperMix for qPCR	Vazyme	Cat#R323-01
AceQ Universal SYBR qPCR Master Mix	Vazyme	Cat#Q511-02
Masson's Trichrome Stain Kit	Solarbio	Cat# G1340
Hematoxylin-Eosin/HE Staining Kit	Solarbio	Cat# G1120
RNeasy Micro kit	Qiagen	Cat#74004
Chromatin-Prep-Kit	Millipore	Cat#MM_NF-17-10461
HiSens-Chromatin-Immunoprecipitation-Kit	Millipore	Cat#MM_NF-17-375
M-PER™ Mammalian Protein Extraction Reagent	Thermo Scientific	Cat#78505
Oligonucleotides		
Mouse Ezh2 siRNA	Origene	SR419773
RT-qPCR primers	Sangon Biotech	appendix table 2
ChIP-qPCR primers	Sangon Biotech	appendix table 3
Software and Algorithms		
GraphPad Prism software v7.03	GraphPad	https://www.graphpad.com/scientific-software/ prism/; RRID:SCR_002798
Image-Pro Plus 7.0	Media Cybernetics	https://www.mediacy.com/imageproplus; RRID:SCR_007369

Primers for quantitative expression analysis of RT-PCR

Gene name		Primer sequence
Ezh2	Forward	AAAGAACTCACGGAGCAGCA
	Reverse	GGAAGGGATGTAGGAAGCAGT
Suz12	Forward	AGCAGTGGCCACAATCGTCTCTAT
	Reverse	TGGAGGTTCCACAGCTTCATCACT
Eed	Forward	ATGCTGTCAGTATTGAGAGTGGC
	Reverse	GAGGCTGTTCACACATTTGAAAG
Ezh1	Forward	CCAGACTGCCAGAATCGCTTT
	Reverse	CAGGTGCTTTTTGAGGCCA
Gapdh	Forward	TCCCACTCTTCCACCTTCGATGC
	Reverse	GGGTCTGGGATGGAAATTGTGAG
Sox2	Forward	TAGAGCTAGACTCCGGGCGATGA
	Reverse	TTGCCTTAAACAAGACCACGAAA
Amelogenin	Forward	TTCAGCCTCATCACCACCTT
	Reverse	AGGGATGTTTGGCTGATGGT
Shh	Forward	CTGGCCAGATGTTTTCTGGT
	Reverse	GATGTCGGGGTTGTAATTGG
Gli1	Forward	CCTTTAGCAATGCCAGTGACC
	Reverse	GAGCGAGCTGGGATCTGTGTAG
Gli2	Forward	AGCTCCACACACCCGCAACA
	Reverse	TGCAGCTGGCTCAGCATCGT
Gli3	Forward	CAACCACAGCCCTTGCTTTGC
	Reverse	GGCCCACCCGAGCTATAGTTG

Primers used for ChIP-qPCR

Gene name		Primer sequence
Shh-Rg1	Forward	CTGTTTTCACAACCCCGT
	Reverse	GCTGCTTCCCCCTTACTT
Shh-Rg2	Forward	ACCCACCAAGCACACA
	Reverse	GCACCACATTCAAGCC
Shh-Rg3	Forward	TGTGAGTAGGATGGGAGAA
	Reverse	CAGACTGAAGGTGTTAGGG
Shh-Rg4	Forward	GAATCTGTACTTGGTCCT
	Reverse	GCTTTTGAGTTAGTGTGA
Shh-Rg5	Forward	CAGCAGGAGGAATAGC
	Reverse	TCCCAGACACCAGACA
Shh-Rg6	Forward	GGCTTAGGGCTTCTCCTTGGCT

	Reverse	GGCTCTTTGGCTCCTCCTTGCT
Sox2	Forward	TTTAGGGTAAGGTACTGGGAAGG
	Reverse	GAGCCCGGGAAATTCTTTTA
